# CLEMD, a circuit-level electrical measurements dataset for electrical energy management

**DOI:** 10.1038/s41597-024-03433-7

**Published:** 2024-06-06

**Authors:** Omar Al-Khadher, Azharudin Mukhtaruddin, Fakroul Ridzuan Hashim, Muhammad Mokhzaini Azizan, Hussin Mamat, Ahmed Aqlan

**Affiliations:** 1 Faculty of Engineering, Universiti Pertahanan Nasional Malaysia, 57000 Sungai Besi, Kuala Lumpur, Malaysia; 2 Faculty of Engineering and Built Environment, Universiti Sains Islam Malaysia, 71800 Bandar Baru Nilai, Nilai, Negeri Sembilan Malaysia; 3 School of Aerospace Engineering, Universiti Sains Malaysia, Engineering Campus, 14300 Nibong Tebal, Penang Malaysia; 4 Networks and Communications Unit, Northern Border University, 91431 Arar, Saudi Arabia

**Keywords:** Energy management, Energy supply and demand

## Abstract

Enhancing energy efficiency in commercial buildings is crucial for reducing energy consumption. Achieving this goal requires careful monitoring and analysis of the energy usage patterns exhibited by different devices. Nonetheless, gathering data from individual appliances in commercial buildings presents difficulties due to the large number of appliances, complex installations, and costs. This paper presents the Circuits-Level Electrical Measurements Dataset (CLEMD). The measurement was conducted at the main switchboard to a set of distribution boards instead of measuring at the individual loads. The data is gathered from an institutional setting. It consists of 42 records of vital electrical parameters including voltage, current, frequency, real power, reactive power, apparent power, power factor, and odd harmonics for electrical currents. The device deployed in the measurement were industry-grade and had a high sampling rate of 200 kHz. The measurements were done over a 40-day period, from September 16 2023 to October 25 2023. CLEMD is the first Malaysian public dataset on circuit-level electricity consumption and offers analysis opportunities in different research areas such as electricity load disaggregation at circuit level, circuit identification, load profile forecasting, and pattern recognition.

## Background & Summary

Buildings are responsible for 40% of total consumption of electrical energy and 20% of total CO2 emissions. Studies have demonstrated that approximately 30% of this consumption is wasted due to sub-optimal operation, malfunctioning of building system equipment or the behaviour of consumers^1^. Comprehending the utilization of energy within commercial buildings is essential for effective energy control. Yet, the lack of detailed submetering frequently obstructs our ability to discern how energy is distributed and utilized. Detailed data with high precision can aid experts in spotting irregularities, establishing energy standards, and carrying out continual commissioning^2,3^.

Enhancing energy efficiency in commercial buildings is of utmost importance for decreasing energy usage. To accomplish this, it is vital to measure and examine the power consumption patterns of various devices. Depending on the specific objective, one might want to gauge the overall energy use, the schedules of device operation, power consumption at specific intervals, or electrical system reliability^3,4^. The repetitive demand patterns in commercial buildings, driven by factors such as shift work and recurring tasks, offer a great opportunity for precise power forecasts. In recent years, advancements in machine learning, artificial intelligence, and data analytics have revolutionized load forecasting techniques, enabling utilities to leverage large volumes of data and complex models to improve forecast accuracy and reliability. Moreover, the proliferation of smart meters, IoT devices, and grid sensors has facilitated the collection of high-resolution data, enhancing the granularity and timeliness of load forecasts^5–9^. The introduction of intelligent meters in these buildings represents a significant step forward in reducing energy consumption and improving operational efficiency.

Smart meters are used to measure the parameters of appliances such as voltage, current, frequency, and power in residential buildings^10–12^. However, in commercial buildings, collecting data from individual appliances poses challenges due to the sheer volume of appliances, intricate installation processes, and associated costs^2,13,14^. The effort to undertake such measurements is not easy. It entails acquiring budgetary allocations, procuring appropriate measuring devices, selecting suitable sites, obtaining necessary permissions, setting up the devices, monitoring their health regularly (typically twice daily), transferring the collected data, and subsequently cleaning and processing it for analysis. To support energy management in buildings, non-intrusive load monitoring (NILM) is employed to break down aggregate consumption data into the contributions of individual devices within a specific structure^15^. The NILM technique emerged in the early 1980s by Hart^16^ and remains as a significant research area of interest today^17–24^. The development of this technique concentrates on the collected data from the appliances and the main meter. Therefore, public datasets are published to support the search for the NILM technique.

Recently, publicly accessible datasets for NILM have been created to facilitate the examination of energy disaggregation and the identification of consumer appliance loads. They enable researchers to replicate prior studies and improve upon their findings. Most datasets focus on data collection of appliances in residential buildings^25–39^, where the measurement resolution is limited to 1 Hz due to the expensive nature of high-frequency sampling. However, sampling frequency is critical in determining the fidelity and accuracy of the digitized signal. It ensures that the original analog signal can be accurately reconstructed from its digital representation^40^. The harmonics at the higher order can be measured at higher sampling frequencies which are difficult to be captured by using low frequencies. Despite differences in sampling frequencies in the majority of datasets, the data interval is 1 second. It is rare to find publicly accessible datasets that have been collected from commercial buildings, such as I-BLEND^3^, BLOND^41^, CREAM^42^, CU-BEMS^43^, and datasets on South manufacturing factories’ electricity consumption^44^.

All NILM datasets primarily target appliances, but NILM applications extend beyond these to include photovoltaics (PVs), electric vehicles (EVs), wind turbines, and smart grids^45^. Additionally, NILM datasets can be used in other fields such as load forecasting (LF), pattern recognition, and event detection. The circuit-level electrical measurements dataset (CLEMD)^46^ focuses on the circuit-level rather than the appliance or equipment level to collect the data.

Data collection involves a diverse range of methods for parameter measurement, including the use of smart meters, wireless sensors, plug load monitors (or wireless plugs), current transformers (CTs), oscilloscope probes, current clamps, and power quality analysers. Table 1 displays a variety of datasets that employ different measurement methods, each with their own characteristics in terms of resolution, duration, and parameters measured.

**Table 1:** Overview of the details in public datasets in terms of resolution, period, measurement method, and parameters.

Dataset	Resolution		Duration	Method	Parameters
	Aggregate	Appliance			
Kim *et al.*^44^	—	—	7 months	Smart meters	P
CU-BEMS^43^	—	—	18 months	Smart meters	P, E
REFIT^30^	8 s	8 s	2 years	Wireless sensors	P
AMPds2^28^	1 min	1 min	2 years	Wireless sensors	V, I, P, Q, S, pf, f
DEDDIAG^37^	1 Hz	1 Hz	3.5 years	Smart meters and wireless plugs	P
ENERTALK^31^	15 Hz	15 Hz	29–122 days	smart meters, wireless plugs, and current transformer clamps	P, Q
BLOND-50^41^	50 kHz	6400 Hz	213 days	wireless sensors and wireless plugs	Vrms, Irms, P, Q, S, pf, f
BLOND-250^41^	250 kHz	50 kHz	50 days		
REDD^21^	15 kHz, 1 Hz	1 Hz	119 days	CT, oscilloscope probe, and wireless plugs	V, I, P
UK-DALE^34^	16 kHz	1/6 Hz	655 days	CT and plug monitor	V, I, P, Q, S
PLAID-II^54^	30 kHz	30 kHz	—	Oscilloscope probe and current clamps	V, I
IDEAL^32^	1 Hz	—	22 months	wireless sensors and current clamps	P, S
WHITED^35^	44.1 kHz	—	—	AC-AC transformer and current clamps	V, I
CLEMD^46^	200 kHz	200 kHz	40 days	power quality analysers	Vrms, Irms, P, Q, S, pf, f, current harmonics

In this work, we proposed the CLEMD. The measurement was done at an educational building. Data collected are voltage, current, frequency, real power, reactive power, apparent power, power factor, and electrical current harmonics (odd harmonic only). All of those data were measured and collected using three-phase power quality and energy analysers. In order to expand choices in future analysis, all measurements were done for all three phases plus the neutral.

The distinctive aspect of the CLEMD dataset detailed in this paper is using the current harmonics in the circuit disaggregation, LF, and pattern recognition purposes is important due to the high-order harmonics caused by the appliances in the circuits in commercial buildings. Including the higher harmonic order can allow the researchers to study the effect of this parameter on the circuit-level. The CLEMD concentrates on circuits rather than home appliances to understand the aggregate behaviour of circuits and the individual circuits.

This article is divided into several sections. The methods section outlines the hardware employed for measuring the circuits, the process of data collection, data transfer, the exportation of data, and the types of appliances in the circuits. Following that, the records data section provides accessibility to data and clarifies the types of files, parameters, missing files, and duration of measurement. The technical validation section showcases the quality and technical validity through the application of histogram plots, weekly and daily pattern plots, harmonics box plots, and measured and designed circuits. The usage notes section gives instructions about the file format, while the code availability section clarifies the scripts of the CLEMD at the repository. The objectives of this study can be stated as the following:

- To measure the data at the circuit-level suitable for circuit disaggregation, circuit identification, load profile forecasting, and pattern recognition purposes. The data consists of the measurement of selected electrical parameters for the circuit supplying downstream several circuits. Measurement is also done at selected downstream circuits.
- Electrical parameters available in the dataset are voltage, current, frequency, real power, reactive power, apparent power, power factor, and odd harmonics for current.

## Methods

The following section outlines the hardware employed for measuring the Main Switch Board (MSBs), Sub-Switch Boards (SSBs), Distribution Boards (DBs), and Control Panels (CPs). Additionally, it elucidates the process of data collection, data transfer, and the subsequent exportation of data into two distinct files. Moving forward, the subsequent subsection expounds upon the aggregation of the MSB, SSB, DB, and CP, and identifies the specific DBs that fall outside the purview of these measurements. In the dedicated section focused on individual Distribution Board measurements, a detailed breakdown is provided, specifying the appliances within each MSB, SSB, and DB, ensuring clarity and precision. Concluding the section, the last subsection delves into known issues, addressing any anomalies or challenges encountered during the measurement process.

### Measurements set-up

The Fluke 435-II Power Quality and Energy Analyser was utilized to measure all the electrical circuits and the aggregate power in the building (https://www.fluke.com/en-us/product/electrical-testing/power-quality/434-435#). The analyser has 4 BNC inputs (line 1, line 2, line 3, and neutral line) for current clamps and 5 banana inputs (line 1, line 2, line 3, neutral line, and ground) for voltages. The analyser offers a comprehensive set of input characteristics to accurately measure and analyse various aspects of electrical systems. This analyser provides measurement for both DC voltage and true RMS voltage for AC system, with a typical measurement range of 1 V to 1000 V. The input impedance is 10 MΩ. For current measurements, the analyser supports AC and DC current measurements using clamp-on current probes, with a wide range of current measurement capabilities. The nominal frequency measurement range for an AC system is typically from 40 Hz to 70 Hz, with an accuracy of around ±0.01 Hz.

The analyser can measure various power parameters such as instantaneous power, real power, reactive power, and apparent power. Analysers also allow measurement of power factors, that is both power factor (PF) and displacement power factor (DPF), typically ranging from 0 to 1. With their harmonic measurements, the analyser can determine total harmonic distortion (THD), and harmonics up to the 50th order for both current and voltage. The quantized analog signal values are then converted to digital binary numbers by the analog-to-digital converter (ADC). Each binary number represents the voltage or current level at a specific sampling point. By converting analog signals to digital form, the Fluke 435-II Power Quality and Energy Analysers enable precise and detailed analysis of power quality and energy consumption in electrical systems. The accuracy of the ADC process is essential for reliable measurements and meaningful analysis of electrical parameters.

**Fig. 1:**
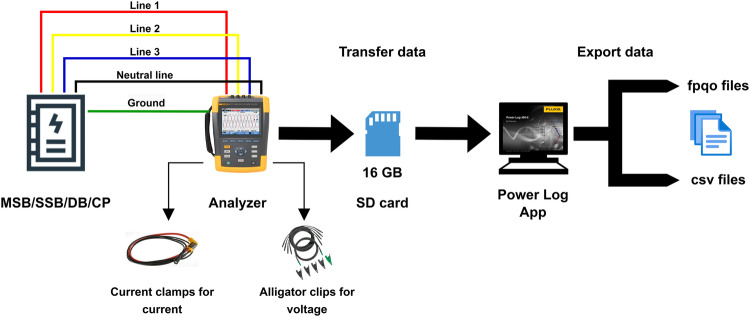
Schematic of data collection, transferring data, and exporting data.

The location of the measurements is located at a low voltage (LV) switch room in the Faculty of Medicine and Defence Health in the Universiti Pertahanan Nasional Malaysia (National Defence University of Malaysia). There were eight analysers used for this measurement. One was designated for the main incoming circuit while the rest were used to measure the seven outgoing circuits. All outgoing circuits had their source taken from the same main incoming circuit. All collected data is based on star connection (line 1, line 2, line 3, and neutral line). The frequency resolution is 200 kHz in the measurements which provide more details about signals. Figure 1 shows the schematic diagram for data collection, transferring, and exporting in different file formats. Once the connections were secured, the next step was to select the specific parameters we wished to monitor before commencing the data recording process. In our chosen dataset, we included crucial metrics such as current, voltage, frequency, real power, reactive power, apparent power, power factor, and current harmonics up to the 15th order.

The analyser recorded the data at one-second intervals over 33 days, meticulously recording observations during both September and October. The decision to focus on one month stems from Malaysia’s tropical climate, which exhibits consistency throughout the year, rendering the selection of any particular month inconsequential to the results. Unlike regions with distinct traditional seasons such as winter, spring, summer, and autumn, Malaysia experiences the influence of only two predominant monsoon seasons. These monsoons, characterized by shifts in rainfall and wind patterns, exert varying impacts on weather conditions and activities across different regions of the country. Nevertheless, Malaysia maintains a relatively stable temperature regime year-round^47–49^. Concerning HVAC, the demand are relatively consistent in the whole year. The data were stored within the analysers' 16 GB SD card. Following the data collection period, it was imperative to pause the recording process and ensure that the data records were safely stored within the SD card. The SD card was required to be retracted from the analyser in order to transfer the data to a computer. To open the data files in the computer, the Power Log application was used which allowed to export of the collected data in various file formats. Exported data were saved in two formats called FPQO (supported by Log Power App) and CSV files. This dual format provides flexibility and versatility in working with the recorded data which allows to conduct in-depth analyses and generate insightful reports as required.

### Aggregate power measurement

The Fluke 435-II has the capability to sample at 200 kS/s on each channel simultaneously, with data available at a rate of 1 Hz for the incoming power supply board. The sampling capability of the device enables the measurement of harmonics up to the 50th order (2.5 kHz). The analyser is connected to four lines: namely, line 1, line 2, line 3, and the neutral line, based on a star connection. The MSB (or aggregate power) receives its supply from the utility company and distributes power to the circuits, which consist of 6 DBs, one EMSB, one SSB, and two CPs. The aggregate power can be determined from the power consumption of appliances connected to the circuits.

To understand the connections of circuits, the primary purpose of the MSB is to receive electrical power from an external source and then distribute that power to various circuits and electrical devices within the building. The MSB contains circuit breakers, switches, or other protective devices that control the flow of electricity to individual circuits. The EMSB serves as the main switchboard for emergency outgoing circuits. During normal operation, the EMSB relies on the MSB for power. However, in emergency situations or during shutdowns, the EMSB becomes the main source for critical outgoing circuits, drawing power from a backup generator to ensure the continuity of essential services. The SSB receives electrical power from the MSB or a higher-level distribution panel. SSBs further split and distribute this power to various subcircuits, equipment in specific areas, or sections within a building. Meanwhile, the DB receives electrical power from the main switchboard or a primary source and then distributes it to multiple individual circuits or subcircuits within a building. These circuits may supply power to outlets, lighting, appliances, machinery, and other electrical loads. Each circuit within a DB is equipped with circuit breakers, fuses, or switches designed to interrupt the flow of electricity in the event of overloads, short circuits, or other electrical faults. The CP is a specialised enclosure housing electrical components, switches, and instruments used to control and monitor various electrical systems and equipment. CPs are commonly found in industrial settings, automation systems, and machinery control applications.

**Fig. 2:**
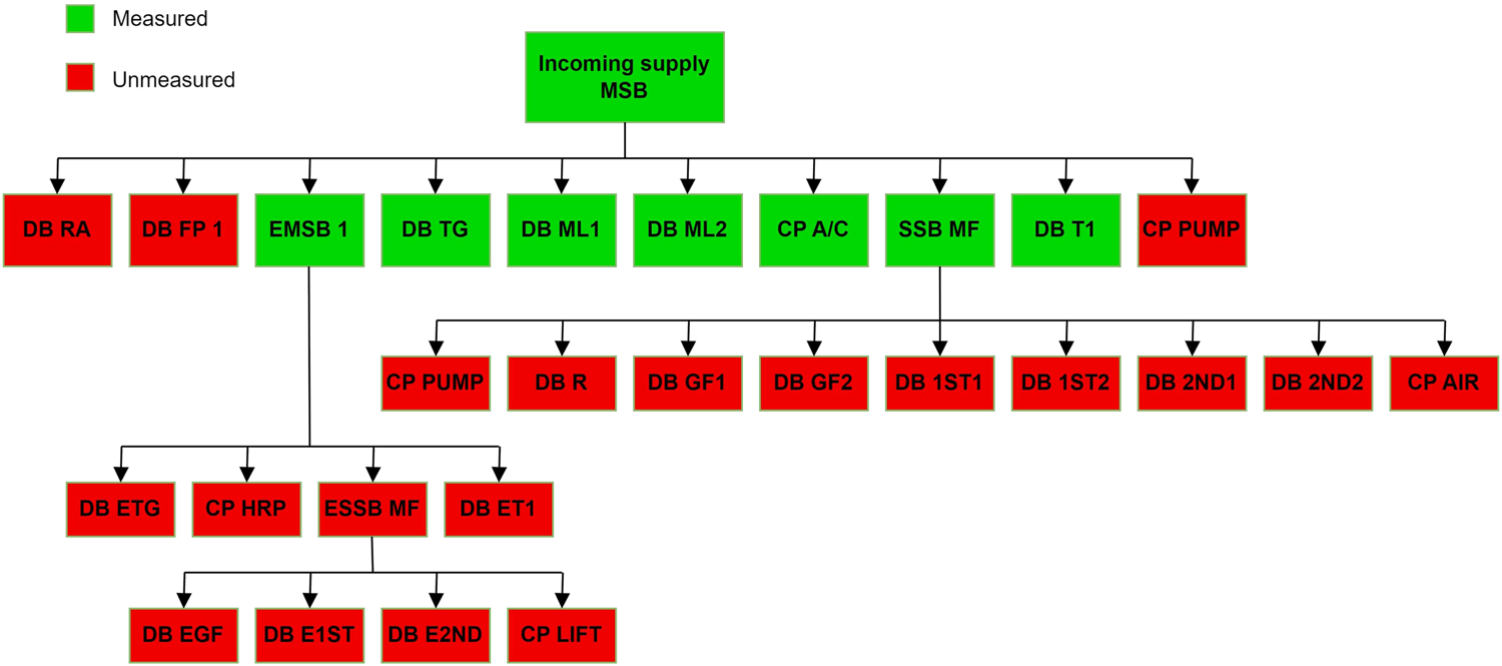
The measured and unmeasured circuits diagram.

Table 2 shows a list of the MSB, SSB, DBs, and CPs connecting to the MSB (aggregate power). It also, describes the total connected load (TCL), maximum demand (MD), and floor location of the loads. The TCL is the sum of the power ratings of all connected electrical devices at a specific moment, whereas the MD is the highest power consumption experienced over a defined period, often used for infrastructure sizing and utility billing purposes. In addition, loads of circuits (MSB, SSB, DB, and CP) are located on various floors in the faculty. Figure 2 presents the diagram of circuits connected to the incoming power supply board. In total, the measurements are focused on four DBs, two MSBs, one SSB, and two CPs that are the most consumed and activated during the day such as DB TG, SSB MF, A/C CP, DB T1, DB ML1, DB ML2, and EMSB 1. The remaining circuits, namely FP 1, PUMP CP, and DB RA, were not measured due to the limited number of analysers available at the time. These circuits were selected due to their infrequent usage and low power usage. The total MD of all three DBs is only 4.68% from the MD of the incoming circuit.

**Table 2:** List of MSB, SSB, CP, and DBs connecting to the aggregate intake.

Name	MSB/SSB/DB/CP	TCL	MD	Floor
TG	DB	10.40 kW	6.87 kW	Ground floor
FP 1		1.65 kW	1.65 kW	Ground floor
ML1		26.10 kW	17.22 kW	Ground floor
ML2		26.10 kW	17.22 kW	Ground floor
T1		11.42 kW	7.65 kW	First floor
RA		8.47 kW	5.95 kW	Roof
MF	SSB	147.38 kW	98.58 kW	Ground, first, second, and roof floors
EMSB 1	MSB	37.79 kW	32.62 kW	Ground, first, and second floors
A/C	CP	50 kW	33.50 kW	Roof
PUMP		0.75 kW	0.75 kW	Roof
Incoming Supply	MSB	301.51 kW	205.81 kW	Ground floor

### Individual circuit measurements

The data interval is provided at 1 Hz for individual circuits, including MSB, SSB, DBs, and CPs, similar to the aggregate power measurement, which is based on three phases and neutral lines. Power consumption varies from one circuit to another due to the different classes of appliances in each circuit. However, Tables 3–5 are presented so that researchers can later understand why certain circuits have specific characteristics in their electrical parameter profiles. The objective of this dataset is to facilitate analysis at the circuit level. Prior knowledge about the types of loads connected to any circuit is not necessary. The data for circuits were measured in the LV switch room to extract parameters including voltage, current, frequency, real power, reactive power, apparent power, power factor, and current harmonics. The subsequent subsections explain the number, location, and names of appliances.

**Table 3:** List of appliances connecting to the DBs TG and T1.

Appliance	DB	Power	Phase	Count	Total power
Recessed downlight	TG	18 W	1	30*2	1080 W
			2	11*2	396 W
			3	10	180 W
Weatherproof fluorescent		18 W	2	2	36 W
			3	2	36 W
Surface downlight		18 W	2	4*2	144 W
Downlight		18 W	2	9*2	324 W
			3	11*2	396 W
LED eyeball		3 W	1	7	21 W
Recessed mounted fluorescent		36 W	2	3*2	216 W
Fluorescent bare channel		18 W	2	2	36 W
Emergency light		8 W	1	3	24 W
			2	5	40 W
			3	9	72 W
Exit sign		13 W	2	5	65 W
			3	3	39 W
Metal halide		150 W	2	4	600 W
Switch socket outlet		250 W	1	8	2000 W
			2	6	1500 W
			3	4	1000 W
Switch socket outlet for A/C		250 W	1	2	500 W
			3	2	500 W
Recessed mounted fluorescent	T1	18 W	1	7*2	252 W
		18 W	2*2	2*2	360 W
		36 W		4*2	
		18 W	2*3	2*2	288 W
		36 W		3*2	
Emergency light		8 W	3	3	24 W
Switch socket outlet		250 W	1	11	2750 W
			2	11	2750 W
			3	11	2750 W
Switch socket outlet for A/C		250 W	1	3	750 W
			2	3	750 W
			3	3	750 W

**Table 4:** List of appliances connecting to the DB ML1 and DB ML2.

Appliance	DB	Power	Phase	Count	Total power
Recessed mounted fluorescent	ML1	36 W	1	5*2	360 W
			2	7*2	504 W
			3	5*2	360 W
Emergency light		8 W	1	6	48 W
Exit sign		13 W	1	6	78 W
Switch socket outlet		250 W	1	28	7000 W
			2	24	6000 W
			3	28	7000 W
Switch socket outlet for A/C		250 W	2	3	750 W
Recessed mounted fluorescent fitting	ML2	36 W	1	5*2	360 W
			2	7*2	504 W
			3	5*2	360 W
Emergency light		8 W	2	6	48 W
Exit sign		13 W	2	6	78 W
Switch socket outlet		250 W	1	28	7000 W
			2	28	7000 W
			3	28	7000 W
Switch socket outlet for A/C		250 W	1	3	750 W

**Table 5:** List of appliances connecting to SSB and DBs from the aggregate EMSB1.

Appliance	MSB	SSB\DB	DB	TCL	MD	Power	Phase	Count
Recessed mounted fluorescent	EMSB1	ESSB MF	EGF	3.4 kW	2.25 kW	36 W	1	5*2
						18 W	2	2*2
						36 W	3	2*2
Recessed downlight						18 W	2	2
							3	8*2
Switch socket outlet						250 W	1	4
							2	4
							3	2
Recessed mounted fluorescent			E1ST	3.01 kW	1.98 kW	18 W	1	5*2
						36 W	3	6*2
Recessed downlight						318 W	1	8*2
							2	8*2
							3	8*2
Switch socket outlet						250 W	1	2
							2	2
							3	2
Recessed mounted fluorescent			E2ND	3.47 kW	2.29 kW	18 W	2*1	6*2
						36 W		4*2
						18 W	2	6*2
						18 W	2*3	2*2
						36 W		2*2
Recessed downlight						18 W	1	2
Switch socket outlet						250 W	1	4
							2	4
							3	2
Lift			CP LIFT	7.6 kW	7.6 kW	—	—	—
Recessed mounted fluorescent		ETG	—	4.59 kW	3.26 kW	36 W	1	9*2
						18 W	2	7*2
						18 W	3	5*2
Recessed downlight						18 W	1	10
							3	4*2
Fluorescent bare channel						36 W	2	9
							3	6
Exhaust fan						—	2	1
							3	1
Switch socket outlet for A/C						250 W	1	4
							2	4
							3	2
Recessed downlight		ET1	—	0.36 kW	0.24 kW	18 W	1	5*2
							2	4*2
LED eyeball						3 W	2	6
S/S/O 13 A						—	2	2
hose reel pump		CP HRP	—	3.7 kW	—	—	—	—

#### DB TG and DB T1

The appliances of DB TG are situated in the administration block of the faculty on the ground floor. Each phase of DB TG includes several appliances, and the number of appliances and their power consumption in each phase varies. Table 3 illustrates a list of appliances connected to DB TG, showing the power rating of each appliance, the phase connection, and the quantity of appliances. The majority of appliances in DB TG are for lighting, and two pairs of lights need to be accounted for. Additionally, the switch socket outlet and switch socket outlet for A/C are connected in the circuit (DB TG).

The electrical appliances associated with DB T1 are located within the administration block of the faculty, specifically on the first floor. Each phase of DB T1 accommodates both lighting and electrical sockets, with each phase exhibiting different power consumption. Table 3 provides a comprehensive inventory of appliances linked to DB T1, detailing pertinent information such as the power rating of each appliance, the respective phase to which they are connected, and the quantity of appliances. Furthermore, it should be noted that within DB T1, the appliances cater to both lighting and electrical sockets, and there are two pairs of lights that require multiplication for accurate power calculations.

#### DB ML1 and DB ML2

The electrical appliances housed in DB ML1 and DB ML2 are situated within the administration block of the faculty on the ground floor. These measurements encompass every phase of DB ML1 and DB ML2, each accommodating a diverse array of appliances, each with its power consumption profile. Table 4 shows a comprehensive list of appliances connected to DB ML1 and DB ML2, along with essential details such as the power rating of each appliance, the specific phase to which they are connected, the respective DB name, and the number of appliances. It is important to note that the appliances within DB ML1 and DB ML2 serve several purposes, with lighting and electrical sockets being the primary uses. Furthermore, there are two pairs of lights within these DBs that require multiplication for precise power calculations

#### A/C CP

The faculty’s air conditioning loads are situated atop the building, and the chosen system for air conditioning is Variable Refrigerant Flow (VRF). VRF is an HVAC technology designed for efficient temperature control across various building areas. This system utilizes refrigerant as the medium for both cooling and heating, enabling adjustable management of refrigerant flow to each indoor unit^50–53^. There are six units of VRF, each with a 10 hp rated power. All of these units are centrally located on the rooftop for ease of maintenance, aesthetics, noise isolation, and to isolate the electrical supplies to these larger machines from other electrical boards. This is to minimize the impact of the starting current of the VRF on the other supply boards. Two units are designed to serve each floor (2 units of VRF for the ground, first, and second floor respectively).

#### EMSB 1

The appliances associated with EMSB 1 are distributed across various locations, with EMSB 1 itself situated in the LV switch room on the ground floor. EMSB 1 contains one SSB (ESSB MF), one CP (HRP), and two DBs (ETG and ET1). ETG and ET1 are situated within the administration block of the faculty, specifically on the ground and first floors, respectively. Whereas CP HRP is situated on the ground floor. As shown in Table 5, the DB ETG contains appliances such as recessed-mounted fluorescent lighting, recessed downlights, fluorescent bare channels, exhaust fans, and switch socket outlets designed for air conditioning. DB ET1 contains recessed downlights, LED eyeball lights, and 13-ampere Switch Socket Outlets (S/S/O 13 A).

To begin with, when tallying the appliances within EMSB 1, it is important to note that ESSB MF comprises several DBs that are integral to the overall measurements. Specifically, DBs EGF and CP LIFT are located within the faculty building on the ground floor, while DB E1ST’s appliances are situated on the first floor of the same faculty block, and DB E2ND’s appliances are housed on the second floor of the faculty block. Notably, as depicted in Table 5, all of these DBs contain the same types of appliances, including recessed-mounted fluorescent lighting, recessed downlights, and switch socket outlets, with the exception of CP LIFT. In terms of their electrical characteristics, each of these DBs exhibits varying power consumption profiles. The TCL for each DB falls within the range of 3.47 kW to 7.6 kW, while the MD varies from 1.98 kW to 7.6 kW.

#### SSB MF

The appliances associated with SSB MF are distributed across various locations, with SSB MF itself situated in the LV switch room on the ground floor. SSB MF is more complex than others due to the number of DBs linked to it. SSB MF contains seven DBs and two CPs, with each DB containing several appliances. Table 6 presents the names of DBs and CPs, TCL, MD, and the floors. To provide further explanation about the involved appliances in the measurement, all three phases are considered. The various electrical appliances are housed within the MSB MF. These appliances include recessed-mounted fluorescents, fluorescent bare channels, downlights, recessed downlights, exhaust fans, wall lights, emergency lights, exit signs, bare channels, switch socket outlets, and switch socket outlets designed for air conditioning. These appliances are distributed across several DBs for effective power distribution.

**Table 6:** List of DBs connecting to the aggregate SSB MF.

Distribution board	TCL	MD	Floor
DB GF1	11.51 kW	7.6 kW	Ground floor
DB GF2	13.44 kW	8.87 kW	
DB 1ST1	9.01 kW	5.95 kW	First floor
DB 1ST2	6.62 kW	4.37 kW	
DB 2ND1	10.24 kW	6.75 kW	Second floor
DB 2ND2	14.32 kW	9.45 kW	
CP AIR	74.69 kW	74.69 kW	Roof
CP PUMP	1.5 kW	1.5 kW	
DB R	1.04 kW	0.89 kW	

### Known issues

- A few circuit files are missing during the measurement due to the analyser hanging. Hanging occurs because heat develops in the devices as they run continuously for the whole duration of measurement. Additionally, the environment in the LV switch room is not cool enough for the devices.
- The analyser may record negative signs for the power factor of circuits, occurring due to incorrect installation of the device. Therefore, after converting data to a CSV file, pre-processing is necessary to remove the negative signs by using absolute value.

## Data Records

The CLEMD dataset can be freely accessed and downloaded from the figshare^46^. We provide the same frequency for the aggregate and circuits at a frequency of 200 kHz. The raw data of circuits and aggregate power are divided into two file format types named comma-separated value (CSV) and FPQO (supported by Log Power 430-II app). However, the initial file format is FPQO which can be opened by using Power Log software to show the figures of data in subplots and can be downloaded for free (https://www.fluke.com/en-my/support/software-downloads/fluke-powerlog-application-software). The Power Log software can summarize the instrument information, measurements (start time and end time, recording interval, duration, and number of events), recording data that includes the chosen parameters, and scaling (for phases and neutral). In addition, it can be used to show statistics and plots such as line graphs, bar graphs, and histograms. FPQO file cannot open and work with it directly in MATLAB or other coding software.

However, we provide CSV files to facilitate the use and reading of the data in Excel or other software. To convert the files, run the Power Log software and open any FPQO file. After that, click download and select the parameters that are needed to be in the TXT file. Lastly, convert the TXT file to a CSV file. Table 7 shows the distribution of files in the folders extracted for FPQO and CSV format files. The dataset has six folders, each containing 8 files, including CSV files and FPQO files. However, a few files are missing due to the analyser hanging and stopping recording in the middle of the week. From 16 September to 21 September folder, all the format files are provided except the aggregate circuits file. In the next folder, DB ML 2 files are missing from 21 September to 27 September and from 11 October to 18 October. Whereas CP AC files are missing from 21 September to 4 October. EMSB 1 file is missing in the fifth folder from 11 October to 18 October. The other files are not missing and are provided with parameters. The data file includes the date, time, and parameters available in each circuit and aggregate power. The files can be found based on the format here^46^.

**Table 7:** The distribution of files in the folders extracted for FPQO and CSV format files.

File	Folder					
	16 Sep to 21 Sep	21 Sep to 27 Sep	27 Sep to 4 Oct	4 Oct to 11 Oct	11 Oct to 18 Oct	18 Oct to 25 Oct
DB TG	✓	✓	✓	✓	✓	✓
DB T1	✓	✓	✓	✓	✓	✓
DB ML 1	✓	✓	✓	✓	✓	✓
DB ML 2	✓	✗	✓	✓	✗	✓
CP AC	✓	✗	✗	✓	✓	✓
SSB MF	✓	✓	✓	✓	✓	✓
EMSB 1	✓	✓	✓	✓	✗	✓
Aggregate	✗	✓	✓	✓	✓	✓

All CSV files for the weeks contain 519001 samples for six days at a 1-second sampling interval for a total of 40 days, spanning from 16 September 2023 to 25 October 2023. The data is recorded 24 hours per day, starting from 11:55:00 to 00:05:00 per week. Each file has 59 columns indicating the date, time, voltage, current, frequency, real power, reactive power, apparent power, power factor, and odd harmonics for current (up to the 15th order). All circuits and aggregate power are represented in three phases, indicated in the columns of the CSV files. Table 8 explains the parameter columns in CSV files based on a star connection (line 1, line 2, line 3, and neutral line) and illustrates the order of harmonics. The Fluke power quality analyser supports an SD card for data storage. The analyser can record data directly to the SD card, which can then be transferred to a computer for data extraction. Since the measurement interval is 1 second, the measurement cannot span more than one week. Hence, we download the data, transfer it to the analyser, and rerun it for the next week.

**Table 8:** The description of parameters columns in CSV files.

Column	Unit	Variable
Date	—	—
Time	seconds	—
Voltage	V	1, 2, 3, and N
Current	A	1, 2, 3, and N
Frequency	Hz	1, 2, 3, and N
Real power	W	1, 2, and 3
Reactive power	Var	1, 2, and 3
Apparent Power	VA	1, 2, and 3
Power factor	—	1, 2, and 3
Total harmonic distortion (THD)	%	1, 2, 3, and N
Odd current harmonics (H)	%	1, 2, 3, and N

## Technical Validation

In this section, we conduct a technical verification of our dataset to showcase its quality and technical validity. Through the application of histogram plots, weekly and daily pattern plots, harmonics box plots, and measured and designed circuits. Histogram plots show the frequency of real power data points within specific ranges on each circuit and the total in the building. Weekly patterns show the real power data for all circuits, while the daily patterns of current showcase the data of each phase in subplots. Box plots assist in visualising the distribution and spread of odd current harmonics. Measured and designed circuits illustrate the difference in the power to ensure the verification.

### Current patterns

**Fig. 3:**
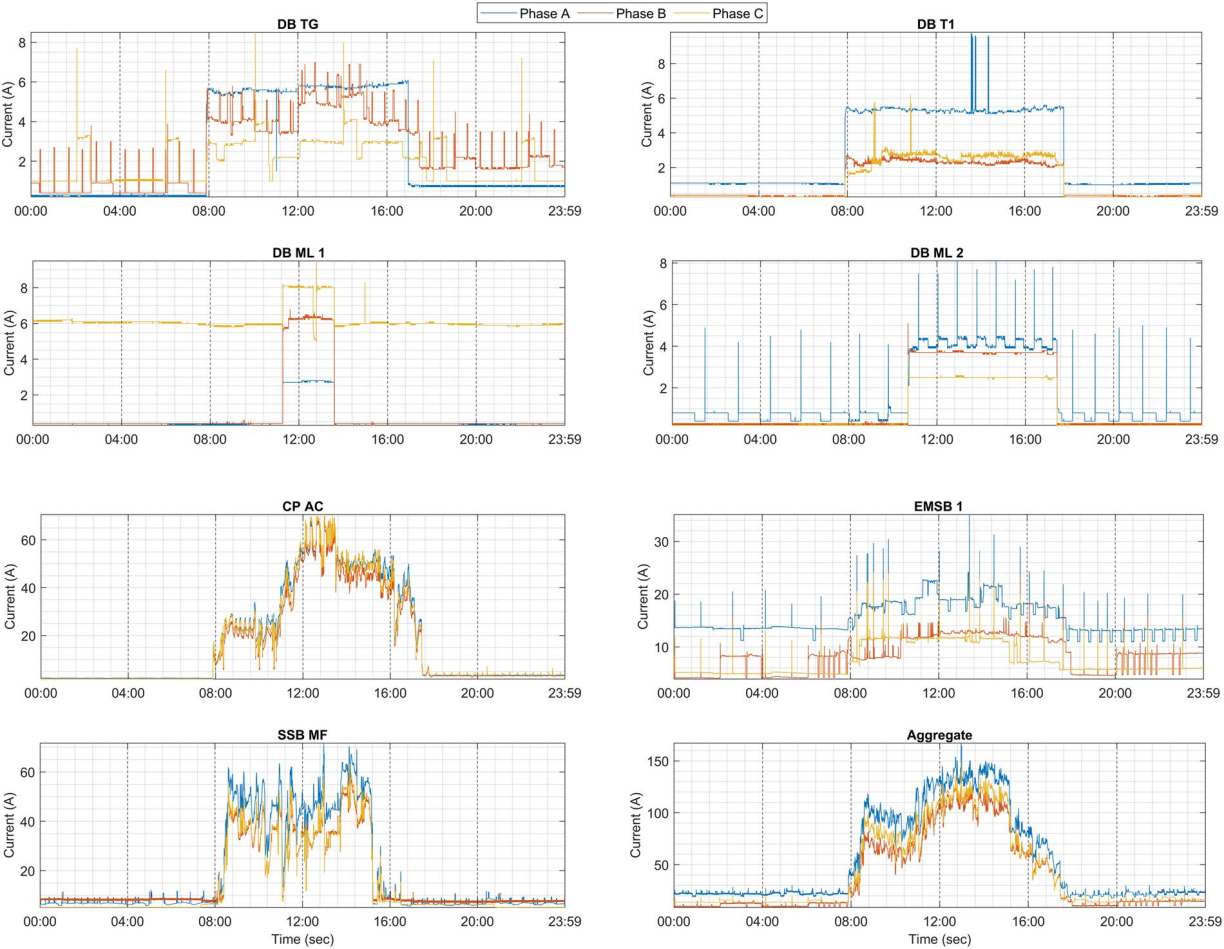
Current patterns of appliances in the circuits based on three phases.

Figure 3 illustrates the daily patterns, showing the current for each circuit based on three phases on Monday. During work hours, the appliances start to be activated roughly between 8:00 am to 6:00 pm. The current patterns vary from one phase to another phase due to the activated appliances in each phase. Except for CP AC, all loads (chillers, induction motors) are activated ensuring that each phase carries an approximately equal amount of current. During non-work hours, some appliances of the circuit are switched off which captures different current patterns for each phase. Moreover, the behaviour of appliances shows the transient and steady states in the circuits.

### Real power data histograms

**Fig. 4:**
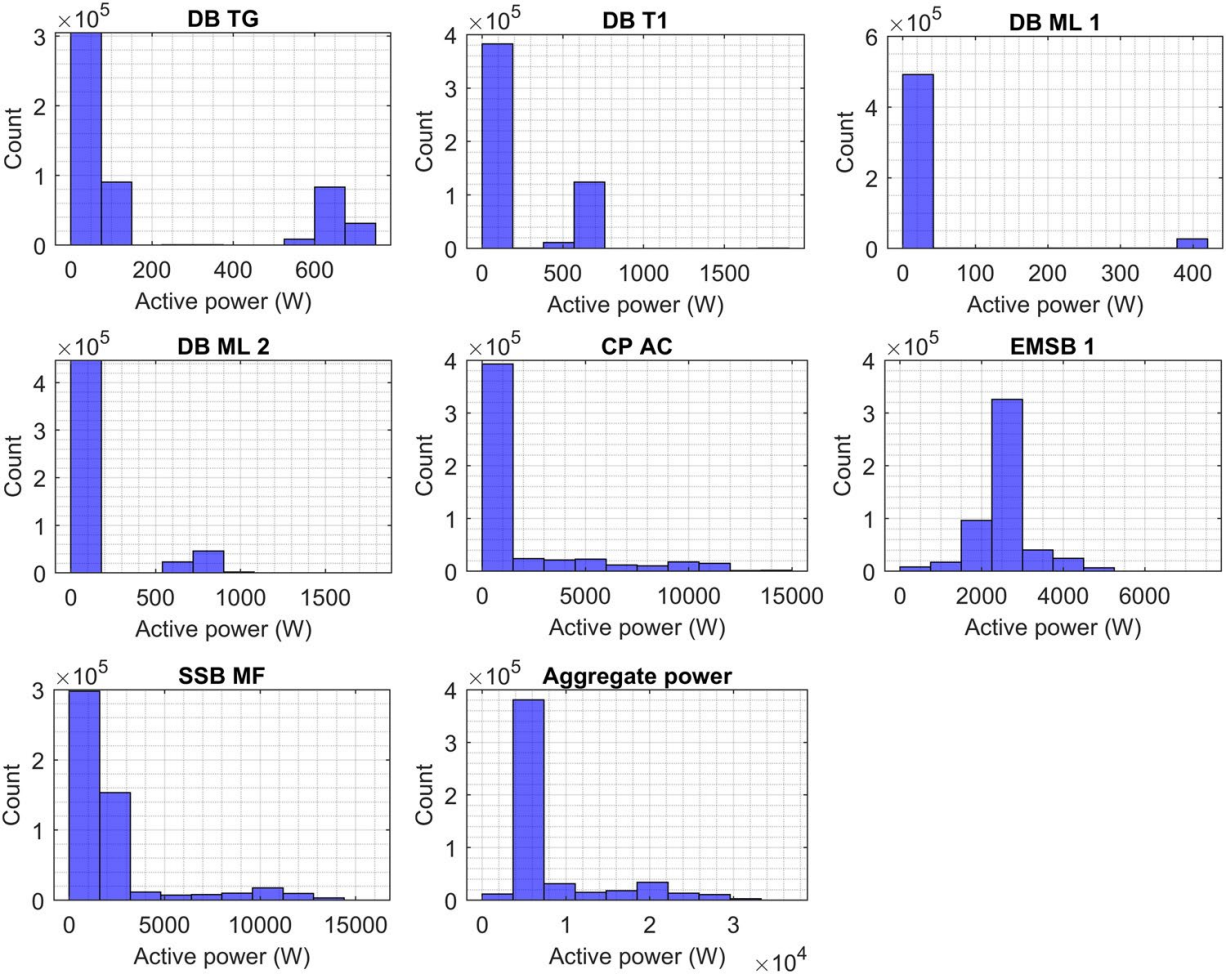
Power consumption histograms of DB T1, DB TG, DB ML 1, DB ML 2, CP AC, EMSB1, SSB MF, and aggregate power based on phase A.

Figure 4 presents the histogram plots of circuits based on phase A, showing the consumed power of DB T1, DB TG, DB ML 1, DB ML 2, CP AC, EMSB1, SSB MF, and aggregate power. As shown the power consumption of DB TG and DB ML 1 are the lowest among the circuits accounting for between 0 and 0.75 kW, and 0 to 0.4 kW, respectively. DB T1 and DB ML 2 showed the amount of power consumption between 0 and 1.6 kW. The highest frequency of power consumption events is observed in the initial bin for DB TG, DB T1, DB ML 1, and DB ML 2. The rest of the circuits are the most consumed such as CP AC, EMSB1, and SSB MF accounting for 0 to 15 kW, 0 to 6 kW, and 0 to 15 kW, respectively. The power consumption occurrence of CP AC is high roughly at 1.5 kW while EMSB 1 from 2 kW to 3 kW. The power consumption occurrence of SSB MF is scattered from 0 to 3.5 kW. In the entire faculty, the aggregate power accounted for 0 to 35 kW, and the highest frequency of power consumption events is 8 kW.

**Fig. 5:**
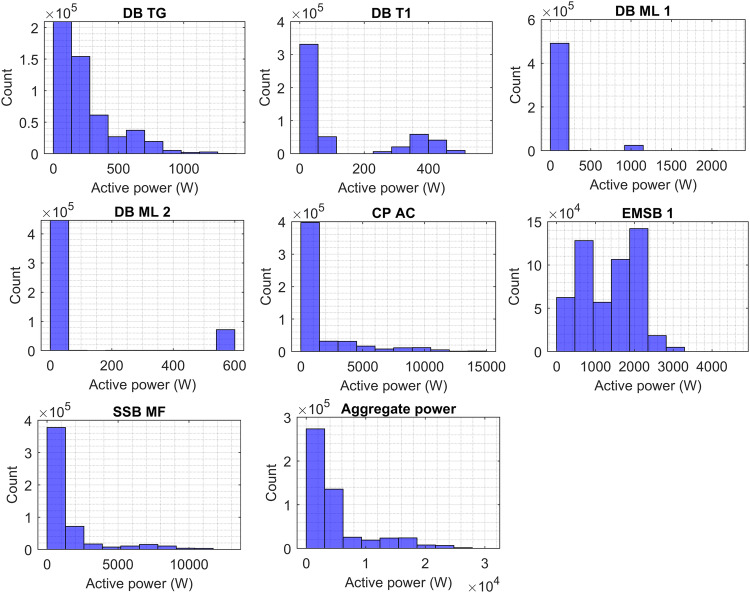
Power consumption histograms of DB T1, DB TG, DB ML 1, DB ML 2, CP AC, EMSB1, SSB MF, and aggregate power based on phase B.

Figure 5 depicts the histogram plots of circuits, showing the consumed power based on phase B. As shown the power consumption of DB TG and DB ML 1 vary in phase B which accounted for between 0 and 1.4 kW, and 0 to 2 kW, respectively. DB T1 and DB ML 2 illustrated the amount of power consumption between 0 and 0.5 kW and 0 to 0.6 kW. The highest frequency of power consumption events is observed in the first bin for DB T1, DB ML 1, and DB ML 2, whereas DB TG is scattered from 0 to 0.3 kW. The power consumption of EMSB1 accounted for 0 to 4 kW, while SSB MF represented 0 to 13 kW. The power consumption occurrence of SSB MF is high roughly at 1.5 kW while EMSB 1 from 0 kW to 2 kW. However, CP AC still consumed the same power consumption accounting for 0 to 15 kW and the same number of power consumption occurrences. The aggregate power consumed from 0 to 30 kW.

**Fig. 6:**
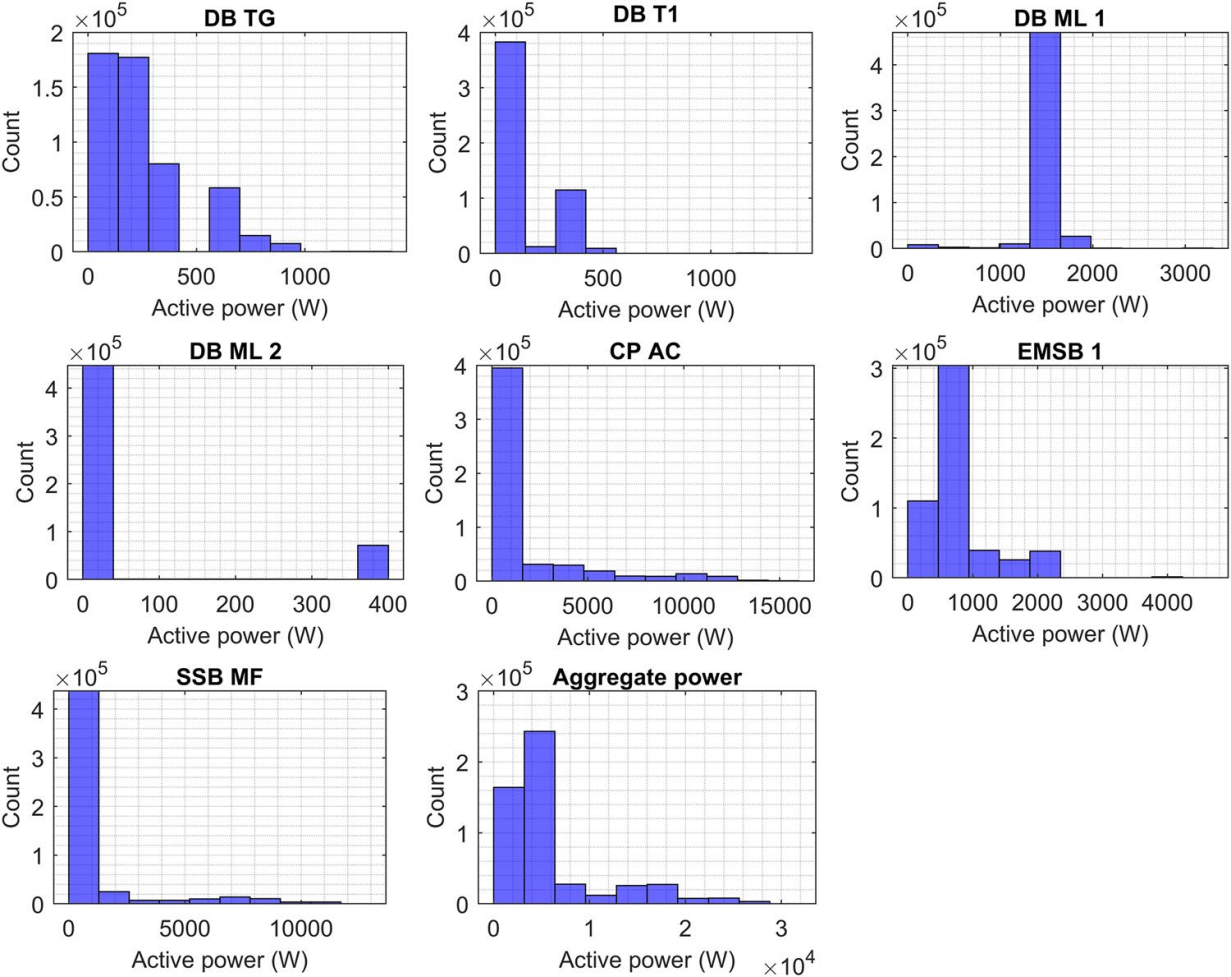
Power consumption histograms of DB T1, DB TG, DB ML 1, DB ML 2, CP AC, EMSB1, SSB MF, and aggregate power based on phase C.

Figure 6 illustrates the histogram plots of circuits, showing the consumed power based on phase C. As shown DB TG and DB T1 consumed between 0 and 1 kW while DB ML 2 accounted for 0 to 0.4 kW which represents the lowest in phase C. DB ML 1 shows a long consumption during the week from 1 kW to 2 kW. The power consumption of EMSB1 accounted for 0 to 4 kW, and SSB MF represented 0 to 13 kW. CP AC still consumed the same power consumption accounting for 0 to 15 kW. The aggregate power consumed from 0 to 30 kW.

### Weekly power patterns

**Fig. 7:**
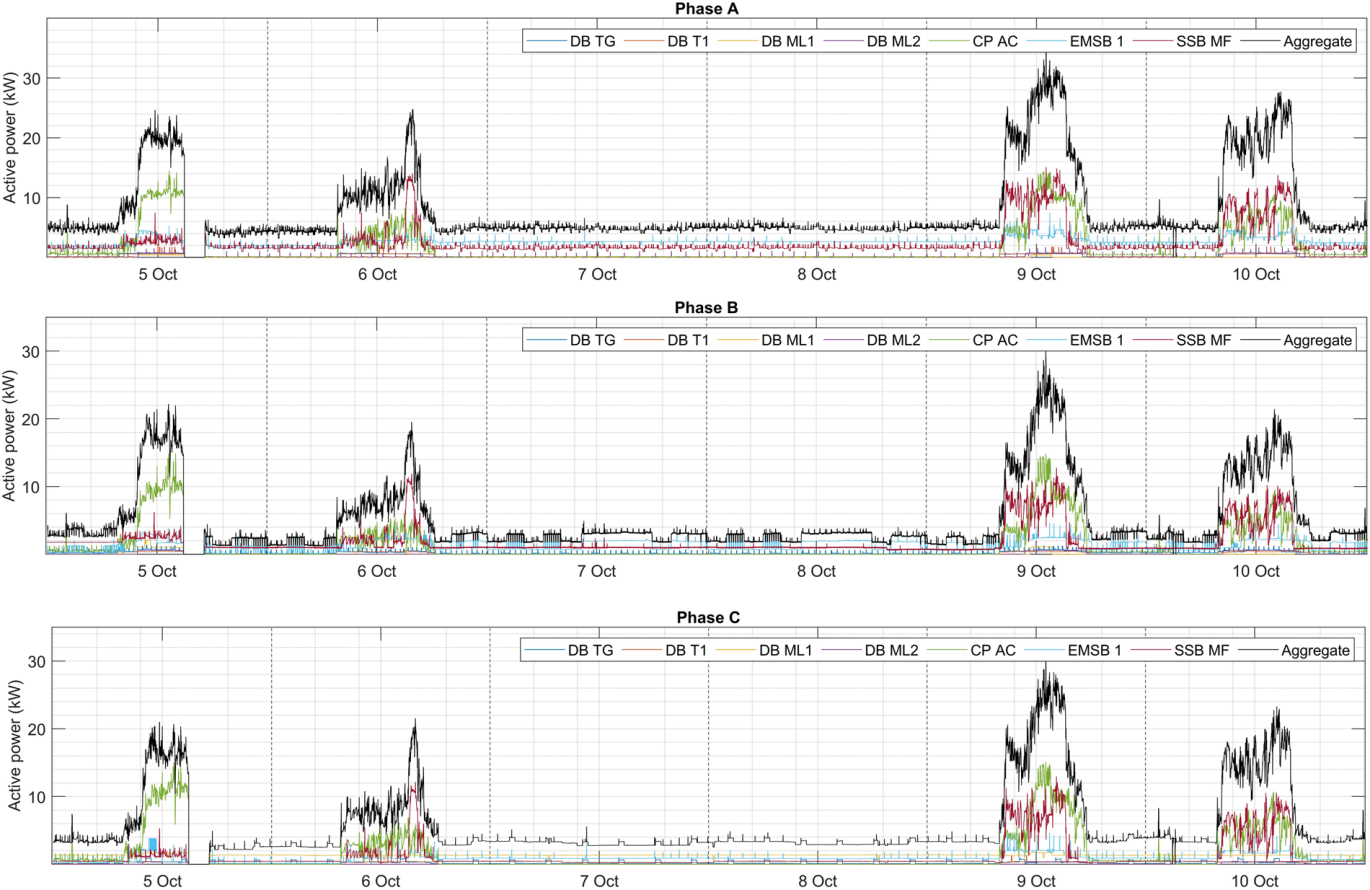
Appliances power consumption in the circuits based on three phases during six days period.

Figure 7 presents the electricity consumption patterns of DB TG, DB T1, DB ML 1, DB ML 2, CP AC, EMSB 1, SSB MF, and aggregate power based on three phases, during a period of six days from 5 to 10 of October. As shown, the circuits highly consumed power during the weekdays (5, 6, 9, and 10 of October), while on the weekends (7 October and 8 October), the power consumption is low due to no use of any of the facilities in the faculty such as classrooms and laboratories. During the weekends, a small amount of power is consumed by emergency lights, exit signs, and several lights which are essential to be on. Also, the electricity consumption ranges from the morning to the evening during work hours on the weekdays. The CP AC, SSB MF, and EMSB 1 circuits are the most consumed in the faculty. CP AC depends on the demand in the building to spread the cool air which consumes more power if necessary. The high electricity consumption of SSB MF and EMSB 1 is because of having several other DBs that are distributed and connected to the appliances. During the weekends, the power consumption may vary in phase A, phase B, and even phase C due to the use of several appliances in specific phase such as SSB MF and EMSB 1 in phase A and B. Whereas, in phase C, all appliances are almost turned off. The aggregate power consumption is proportional to the consumption of circuits based on three phases, which show the validity of data can be used for aggregate circuit and individual circuits.

### Odd harmonics bar graphs

**Fig. 8:**
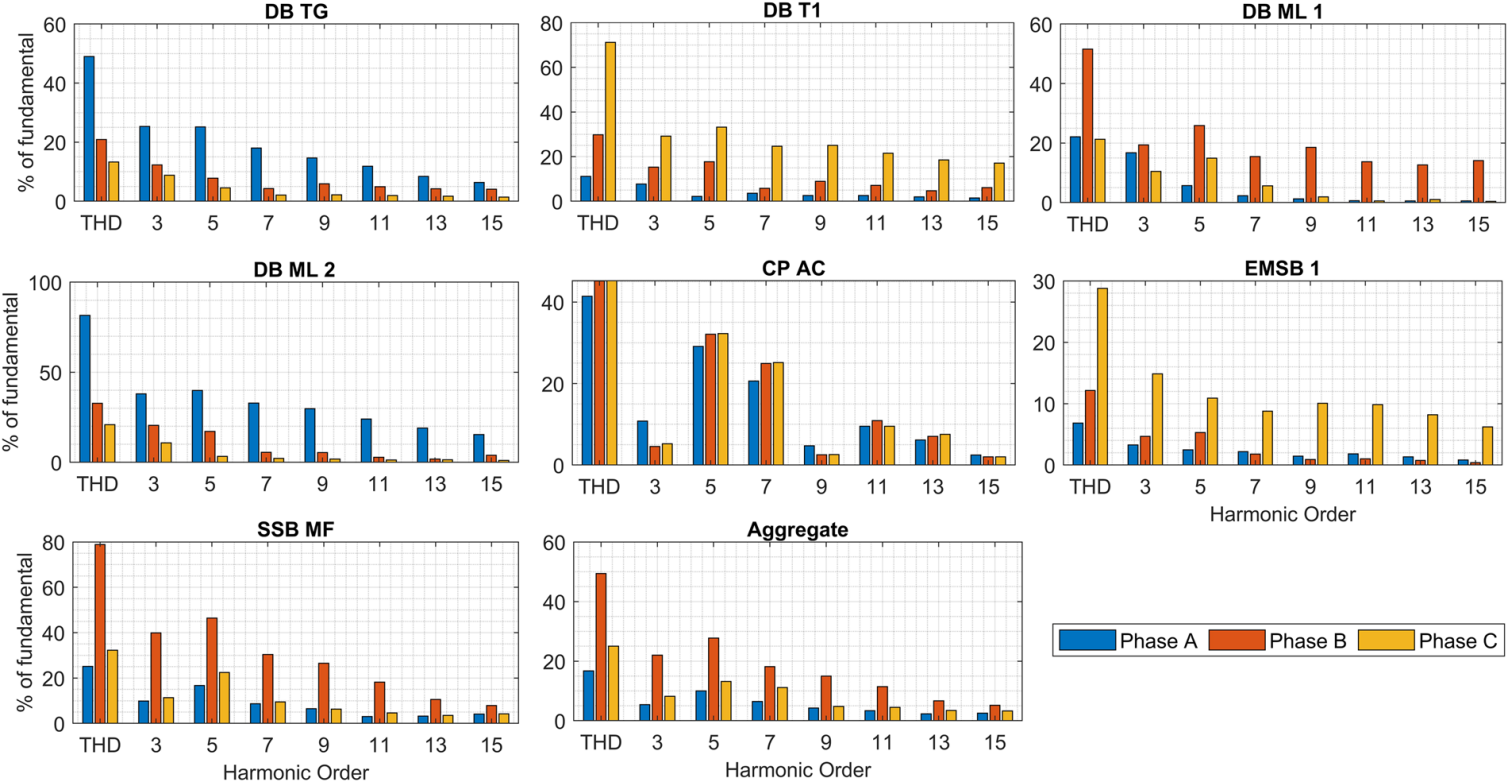
Odd current harmonics bar graphs for circuits based on three phases.

Non-linear or distorting loads are electrical devices or equipment that draw non-sinusoidal current waveforms from the power supply. Non-linear loads are a common source of harmonic distortion in electrical systems. Non-linear loads are computers, laptops, LED lights, metal halide lamps, HVAC systems, industrial machinery, and lifts. Figure 8 shows odd current harmonics and total harmonic distortion for each circuit based on three phases. As shown, odd harmonics are multiples of the fundamental frequency that have odd-numbered integer values. The frequency of each odd harmonic is 50 Hz (fundamental), 150 Hz (3rd harmonic), 250 Hz (5th Harmonic), 350 Hz (7th harmonic), 450 Hz (9th Harmonic),550 Hz (11th harmonic), 650 Hz (13th Harmonic), and 750 Hz (15th Harmonic).

Based on each phase, EMSB 1 represents the lowest THD accounting for 6.84% (phase A) and 12.13 (phase B) while DB TG represents 13.26% (phase C). On the contrary, DB ML 2, SSB MF, and DB T1 show a higher THD percentage at 81.6% (phase A), 78.85% (phase B), and 71.21% (phase C), respectively. Higher-order harmonics show a higher THD percentage indicating more distortion in the waveform due to the presence of significant harmonic components. The odd harmonics vary in each phase because of the different numbers of activated appliances which lead to a decline and increase in the harmonics in all phases differently. However, CP AC almost has the same odd harmonics in each phase due to three-phase loads that work at the same time together. As known, the higher-order harmonics such as 3rd, 5th, 7th, and 9th have the highest harmonics gradually. In CP AC, th5, th7, and 11th are higher than the 3rd and 9th harmonics because of the connection of three-phase loads (star-delta). Unique harmonics characteristics in each circuit give an advantage to load disaggregation, LF, and pattern recognition which can be used to classify the circuits.

### Measured and designed circuits

It is a customary in Malaysia to use maximum demand (MD) in order to estimate the maximum load (real power) that is going to be drawn from a distribution board (DB). This information is also used to determine the size of circuit breaker an eventually the size of cable. To estimate MD, one must first calculate all the individual electrical loads that are drawing their power from the DB. This process is referred to as total connected load (TCL). Following this, it is necessary to estimate the diversity factor (DF). DF is a factor that represents what is the probability that all loads will be switched on at the same time. DF takes the value of 0 to 1. So, The MD is calculated by the formula

1 $$MD=DF\,\ast \,TCL$$

**Fig. 9:**
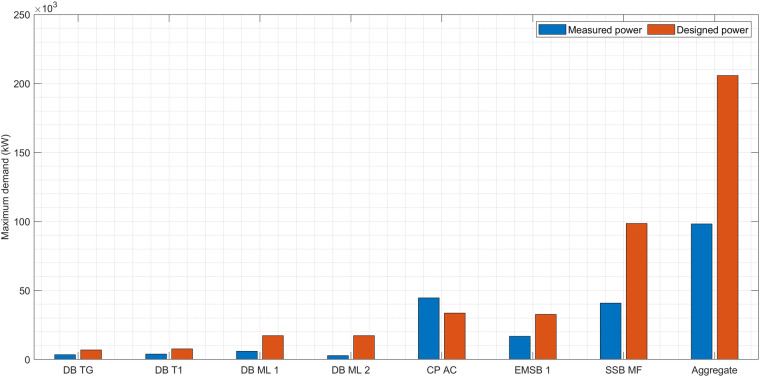
The maximum demand for measured and designed circuits.

Table 2 presents the MD for designed circuits. The accuracy of the designed circuits, which represents the anticipated electrical demand based on engineering calculations and specifications, is crucial for ensuring the adequacy and reliability of the DB. By conducting a comparison with measured circuits obtained through on-site measurements to ensure the verification of data. Figure 9 presents a comparison of maximum demand between designed and measured circuits. To calculate the MD of the measured circuit, the maximum value for each line is identified and gathered. As shown, all the power of measured circuits is lower than that of the designed circuits, which is relatively reasonable except for CP AC. To illustrate, most diversity factors (DF) are estimated, which is not accurate. Table 2 shows that the total connected load (TCL) of CP AC is 50 kW, while the MD is estimated to be 33.5 kW. This, in return, gives a DF of 0.67. However, a DF is very low for a DB that supplies power to an air-conditioning system. Usually, for DBs that supply power to air-conditioning systems, the DF is 1. In this case, the actual maximum power measured of CP AC is 44.62 kW, which is not far from the TCL of 50 kW. The only issue is that the designer of this system had chosen a very low DF. Hence, the comparison between the measured and designed values for CP AC is taken from TCL due to the low DF.

The actual installed cables and circuit breakers on the DB AC are sufficient to carry the actual power (44 kW). From the actual power, the current will be around 75 A (considering 3-phase, 400 V, power factor 0.85). Therefore, the cable should be equal to or larger than 16 mm sq (this cable can carry 92 A - PVC cable). If the designed MD of 33.5 kW, the current would be 56 A. Thus, the smallest cable that can carry 56 A is 10 mm sq (current carrying capacity, or ampacity = 72 A). However, the designer must take into account what is called derating due to temperature and other reasons, as well as voltage drop. So, the installed cable should be 25 mm sq, which can carry about 125 A.

## Usage Notes

Our dataset provides CSV files that have a straightforward file format, using plain text with commas to separate data fields. This simplicity makes it easy to create, read, and write CSV files programmatically, even in low-level programming languages. All CSV files are in several folders. The folders are separated into six based on the date of data collection. The size of each CSV file roughly from 101–164 MB. The data may take more time when it is opened due to the size of the file. Hence, FPQO format can be used in Power Log software (https://www.fluke.com/en-my/support/software-downloads/fluke-powerlog-application-software) to select the necessary parameters and minimize the CSV file size.

## Data Availability

The code of the CLEMD was written in MATLAB. The scripts include four files with all the technical verification. The scripts show histogram plots, daily current patterns, weekly real power patterns, current harmonics bar graphs, measured and designed circuits graph. All the scripts are available at the university’s github repository (https://github.com/CLEMDatabase/CLEMD).
